# Effect of the Intake of Brown Rice for Six Months on the Cognitive Function in Healthy Elderly Persons: A Study Protocol for a Pilot, Non-Randomized Controlled Trial

**DOI:** 10.3390/mps4040078

**Published:** 2021-10-28

**Authors:** Yuji Takano, Keisuke Kokubun, Keiji Saika, Naoki Nishiyama, Yasuyuki Taki

**Affiliations:** 1 Smart-Aging Research Center, Tohoku University, Sendai 980-8575, Japan; yuji.takano.a6@tohoku.ac.jp (Y.T.); yasuyuki.taki.c7@tohoku.ac.jp (Y.T.); 2 Department of Psychology, University of Human Environments, Matsuyama 790-0825, Japan; 3 Toyo Rice Corporation, Tokyo 104-0061, Japan; somu@toyo-rice.jp (K.S.); nishiyama@toyo-rice.jp (N.N.); 4 Research Institute for Agricultural and Life Sciences, Tokyo University of Agriculture, Tokyo 156-8502, Japan; 5 Department of Aging Research and Geriatric Medicine, Institute of Development, Aging and Cancer, Tohoku University, Sendai 980-8575, Japan

**Keywords:** brown rice, cognitive function, elderly, Japanese

## Abstract

The nutritional components of brown rice have been reported to be effective against diabetes mellitus. Recent animal studies have suggested that it is also effective in maintaining cognitive function. Therefore, in this study, we examined the effect of a brown rice diet on cognitive function in individuals aged over 60 years. The study participants were recruited from a pool of individuals aged ≥60 years who were using elderly care facilities. The participants were provided with four servings of brown or white rice per week for 6 months, and their cognitive function was measured before and after the intervention period. Prior to the intervention, participants tasted the white and brown rice to determine which type they would like to be offered over the 6-month period. Since rice is the staple food of the participants in this study, they were allowed to decide whether they wanted to eat white or brown rice.

## 1. Introduction

Rice is commonly consumed as a staple food by approximately 60% of the world’s population [1]. Rice is cultivated in more than 100 countries, with Asia accounting for approximately 90% of the world’s total production and consumption [2]. Rice also plays a major role in global food security because it is the staple food for over half of the world’s population [2]. It could be said that Asia, with more than half of the world’s population and one-third of global poverty, is the center of global food security [3]. Therefore, especially in Asia, attention should be paid not only to the amount but also to the nutrients of rice.

Rice is an excellent source of magnesium, phosphorus, manganese, selenium, iron, folic acid, thiamine, and niacin, and nutrients vary depending on varieties, cultivation methods, and processing methods [4,5]. Rice is classified as white or brown depending on the post-harvest processing, and brown rice is specifically considered to be rich in nutrients with health benefits, and the benefits of each nutrient have been studied. In experiments with mice, gamma oryzanol (GORZ), a nutrient found only in brown rice that enhances insulin secretion and inhibits glucagon hypersecretion, benefitted those with diabetes [6], improved obesity due to high lipid intake [7] and islet dysfunction associated with lipotoxicity [8], and mitigated animal fat overconsumption by reducing endoplasmic reticulum (ER) stress in the hypothalamus [9]. Therefore, GORZ in brown rice is considered to be effective in alleviating the symptoms of diabetes and obesity in humans [10]. In support, in studies of humans, the benefits of brown rice on diabetes [11], obesity [12], and cardiovascular disease [13] were reported, which were consistent with predictions from studies that focused on the specific nutrients found in brown rice. Brown rice is also known for its high phenolic content that promotes human health by reducing oxidative damage, such as phenolic acids and flavonoids [14]. Higher intake of phenolic acids and flavonoids has been associated with cognitive status and function in observational studies of humans [15,16,17,18,19,20]. Specifically, among phenolic acids, ferulic acid, though also found in cereals other than brown rice, is abundantly present in brown rice, and there have been numerous studies on its benefits (see review [1]). Ferulic acid has been shown to have anti-inflammatory [21], antioxidant [22], and anti-diabetic [23] effects and inhibit the accumulation of amyloid-β in the brain [24] in laboratory animal studies. On the other hand, white rice is not a healthy food compared to brown rice because it does not contain bioactive compounds such as minerals and vitamins due to milling and blood sugar level rises quickly after eating. Nevertheless, white rice is consumed more than brown rice because of its ease of cooking and eating [25].

Dietary habits linked to diabetes and obesity are considered to be risk factors for dementia, and the link between dietary habits and dementia has been actively discussed (see review [26]). In recent years, a large cohort study by the UK Biobank suggested that even if people carry genes resulting in high risk factors for dementia, their risk for dementia may be reduced if their diet does not lead to diabetes or obesity [27]. In other words, diet may be important for the prevention of dementia, either indirectly or even directly. Therefore, the present study was designed to investigate the effect of brown rice consumption on the maintenance of cognitive function in the elderly. For that purpose, there is a study on laboratory animals that we should refer to. In mice from the senescence-accelerated mice prone 8 (SAMP8) line, powdered brown rice mixed with a normal diet for 2 months has been shown to reduce the decline in working memory compared to the SAMP8 mice that were fed a diet of white rice powder [28].

On one hand, at the time of writing this manuscript, there were no active intervention studies on human subjects on cognitive ability, except for a few studies [29,30]. For instance, in the study by Uenobe et al. (2019), elderly people living in an elderly welfare facility were divided into two groups, and brown rice or white rice was ingested three meals a day for six months. As a result, brown rice intake improved cognitive function in subjects with low cognitive function at the baseline, suggesting that long-term brown rice intake may help prevent cognitive decline in the elderly [29]. However, since the total number of participants was only 31 and there was only one evaluation scale used for the experiment (Hasegawa Dementia Scale), we thought it is worth testing whether the results could be reproduced when the number of people was increased and another test method was used. Furthermore, given that improvement was seen only in subjects with low cognitive function, we thought it would be valuable to examine how effective it is for elderly people whose cognitive function is maintained at a certain level.

There are also some studies that show white rice is more negatively related to human health than brown rice [31,32,33]. For instance, in an intervention experiment by Saika and Yonei (2021), companies that had newly introduced the brown rice diet decreased medical expenses by 39–40%, indicating that the ingestion of brown rice improved health conditions, decreased disease incident rates, and consequently reduced public medical care expenditures [31]. However, there are also inconsistent reports. A study of Japanese people aged 70 and over found that there was no correlation between a rice-based diet and cognitive function [34]. Moreover, a study of Japanese middle-aged and elderly individuals has shown that a diet centered on rice leads to improved health indicators [35]. These suggest that the nutritional deficiency of white rice can be supplemented by taking nutritious side dishes together, and it is expected that the effect is even greater if nutritious brown rice is ingested in exchange for white rice.

Therefore, in this study, we aimed to clarify the changes in the cognitive function of healthy elderly people by the intake of brown rice, hypothesizing that the brown rice, which contains a large amount of phenolic content and fiber content, helped maintain a higher level of cognitive function than the white rice. The reason for focusing on rice is that it has a great impact on society as it accounts for 20% of the total calorie intake in the world as a staple food [36]. In other words, if the effect of ingesting brown rice, which has more nutrients than white rice, on cognitive function can be clarified, it will promote the spread of dishes centered on brown rice, and will contribute to the health of elderly people around the world, especially those who live in Asia and eat rice as their staple food. However, one of the difficulties associated with the long-term consumption of brown rice diets is the difficulty in digestion [25]. Therefore, in this intervention study, we decided to use brown rice with the outermost wax layer removed. The intervention period was set at 6 months, with measurements of cognitive and psychological functioning primarily conducted before and after the intervention. To control for the effect of nutrient balance from elements other than brown or white rice, we decided to recruit elderly individuals in aged care facilities and elderly day-care facilities where a menu prepared by a dietician was provided.

## 2. Methods and Analysis

### 2.1. Study Design and Objectives

The study was a non-randomized controlled trial; it had a between-subjects design that compared cognitive function before and after the intervention in a group of older adults who consumed brown rice and a group of older adults who consumed white rice, both at four servings per week over a 6-month period. The purpose of this study was to investigate whether long-term consumption of brown rice was more likely to preserve cognitive function than long-term consumption of white rice. Cognitive functioning was assessed using the examination tools of mental state, frontal lobe functioning, daily conversation, and creativity, etc.

### 2.2. Criteria for Inclusion/Exclusion

Participants were recruited from a pool of individuals aged ≥60 years in homes for the elderly and in day-care facilities for the elderly in Japan. Amongst the criteria for participation was the absence of dementia, and written consent was obtained from each participant. In addition, participants had to agree to consume one meal of brown or white rice 4 days a week for 6 months during the study period. Three exclusion criteria were established: (1) participants with scores less than 23 on the Mini-Mental State Examination (MMSE) [37] (suspicion of dementia and Alzheimer’s disease); (2) participants with severe visual or hearing impairments; and (3) participants who had consumed brown rice continuously during the year before the study. On the other hand, people with chronic diseases and/or abnormal weight were not excluded, and these data were used as covariates in the analysis. Although it was not clear whether these covariates eliminate all the complicated effects that can have on the relationship between rice intake and cognitive function, we took larger inclusion criteria following the previous brown/white rice [29,30] and other dietary [38,39] intervention studies of elderly subjects to measure the effect on the diverse population who ingest rice as a staple food.

### 2.3. Rice Used for the Intervention

Brown rice with the wax layer removed and white rice milled from brown rice of the same origin and variety in Japan were used in this study. The rice used in this study was cultivated by “conventional cultivation”, a commonly used cultivation method that uses ordinary amounts of pesticides and fertilizers, for both brown and white rice. In this study, because we focus on the difference in the effect of brown and white rice, and because the cultivation method is known to affect the nutrients of rice [4,5], we adopted a very general cultivation method. The nutritional composition of each rice sample is presented in Table 1. The nutrient analysis was conducted by Japan Food Research Laboratories.

## 3. Protocol

**Figure 1:**
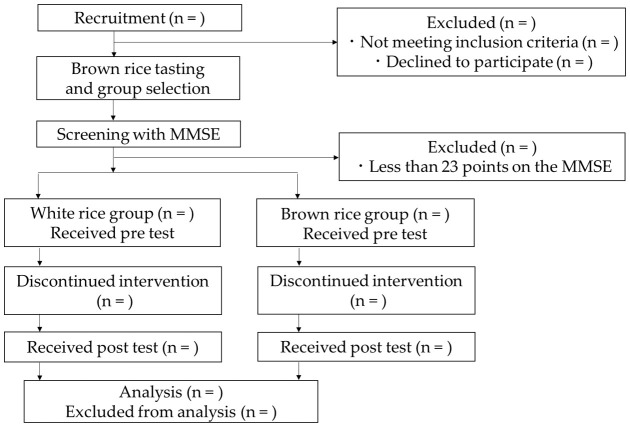
Flowchart of the brown rice intervention study.

The flow chart of this study is shown in Figure 1. The purpose and design of the study were explained to the participants, and their written consent was obtained. The participants were then asked to taste the low-cut brown rice and choose whether they wanted to follow the 6-month white or brown rice intervention.

The participants were then subjected to MMSE [37] testing, and participants with a score below 23 were excluded from the study. Participants with an MMSE score of 23 or higher underwent the Frontal Assessment Battery (FAB) [40] and S-A Creativity Test [41] to assess their cognitive functions. In addition, regarding mental health, the participants completed the General Health Questionnaire 12 (GHQ12) [42] and the visual analog scale questionnaire on current well-being, health, and sleep. Finally, the participant’s fingertip volume pulse wave was recorded in an autonomic test. A staff member who frequently spoke with the participants in the facility was asked to respond to the Conversational Assessment of Neurocognitive Dysfunction (CANDy) [43] to assess the participants’ daily conversation skills. Thereafter, the intervention with the participants by providing the designated brown or white rice was commenced. After 6 months, we performed the same tests as described above. At the end of the study, the participants in the white rice group were asked if they wanted to eat brown rice, and those who agreed were provided with the same amount of brown rice as the brown rice group for 6 months.

## 4. Measurement Items

### 4.1. Mini-Mental State Examination (MMSE)

The MMSE [37] is a 30-point test that assesses the overall cognitive function in the elderly; scores lower than 23 indicate suspected dementia and, together with other test results, medical doctors confirm the diagnosis of dementia. The higher the score, the higher the cognitive function. The test was conducted by a tester in person.

### 4.2. Frontal Assessment Battery (FAB)

The FAB [40] is an 18-point test that measures executive function, i.e., the cognitive function of the frontal lobe, measured by a tester in a face-to-face manner. The higher the score, the higher the cognitive function. The test was also conducted by a tester in person.

### 4.3. S-A Creativity Test

The S-A Creativity Test [41] measures the creativity of how many responses one can give to six open questions. The first two questions asked the participants to answer what an object could be used for, other than general use (e.g., “How do you use a milk bottle?”). The next two questions asked the participants to imagine how a product that exists today will be used in the future (e.g., “What kind of shoes will be used in the future?”). The last two questions were about what would happen if an improbable event occurred (e.g., “What would happen if every human face on Earth was the same?”). Since the writing speed was likely to vary among participants, a tester asked them to answer orally and recorded participants’ responses. For scoring, the scores for the number of responses were added.

### 4.4. Conversational Assessment of Neurocognitive Dysfunction (CANDy)

CANDy [43] is a 15-question scale based on which others rate the characteristics of the daily conversation of the elderly with dementia. This scale has a maximum score of 30 points, with high scores indicating the presence of a variety of conversational features. This score is known to negatively correlate with MMSE scores. Grading was conducted by the staff of the elderly facilities.

### 4.5. General Health Questionnaire 12 (GHQ12)

The GHQ12 [42] is a questionnaire that measures the mental health on a 12-point scale, with high scores indicating poor mental health. The participants completed the questionnaires themselves.

### 4.6. Visual Analog Scale (VAS) on Happiness, Health, and Sleep

The participants were asked to respond to questions about happiness, health, and sleep on the 100-point VAS. The participants completed the questionnaires themselves.

### 4.7. Fingertip Volume Pulse Wave

The fingertip volume pulse wave was measured for 5 min using PowerLab (ADInstruments, Dunedin, New Zealand) with a pulse transducer wrapped around the finger. The measurement was conducted by a tester in person.

### 4.8. Questionnaire on Specific Health Examination (QSHE) and Dietary Variety Score (DVS)

Previous studies have shown that some diseases [44,45,46], overweight [47], physical activities [48,49,50], sleep [51], dietary contents [52,53], supplementation [54], smoking [55], alcohol [56,57] and caffeine [58] consumption, and others [59,60] have a relationship with the cognitive abilities of elderly people. Therefore, information of participants’ past medical/psychiatric history, healthy practices, dietary content other than white and brown rice, etc. was obtained from the person in charge of the facility. For the past medical/psychiatric history and healthy practices including exercise, sleep, drinking, smoking, medicine, etc., we used the questionnaire on specific health examination (QSHE) [61]. QSHE is commonly used in examinations aimed at preventing lifestyle-related diseases and metabolic syndrome in Japan. For the dietary content, we used the questionnaire of dietary variety score (DVS) developed by Kumagai et al. [62]. The DVS is a food-based composite score that is determined by calculating consumption frequencies for 10 food groups, namely, meat, fish/shellfish, eggs, milk, soybean products, green/yellow vegetables, potatoes, fruit, seaweed, and fats/oils, which as a whole constitute a large part of Japanese daily main and side dishes. A score of 1 was given if a food item was consumed every day, otherwise the score was zero. In addition to these, we requested the submission of medical certificates for study participants.

The information obtained from these questionnaires and medical certificates was treated as covariates to control for the effects of past medical/psychiatric history, healthy practices, foods, etc. on cognitive function. Furthermore, there were no particular restrictions on the amount that cannot be measured by these questionnaires, such as the amount of rice, dietary fiber, and polyphenols ingested to prevent the stress felt by the participants by setting restrictions from affecting the research results. In exchange, the related information was obtained from the person in charge of the facility and used as the covariate during analysis. Since the facilities participating in the current study keep a record of the daily meals of the study participants, there is an advantage that such information can be obtained relatively easily. Another strength of this study is that it is unlikely that major nutritional differences will occur among the study participants in a facility because they are served meals using a common menu.

## 5. Sample Size

This study included 76 participants (38 in the brown rice group and 38 in the white rice group). Setting the significance level (α) at 5%, the effect size at 0.6, and the test power (1 − β) at 80%, which are all the “moderate” levels according to Cohen [63], the number of people needed was 34 each for brown and white rice. Assuming a 10% drop rate, we determined that 76 recruits would be needed. However, in this study, the participants chose brown or white rice, so bias may have occurred in the number of people between groups. If a bias occurred, additional recruitment was conducted so that the number difference between the two groups was less than twice as large to prevent the loss of normality in the data.

## 6. Statistical Considerations

We planned to conduct a two-factor analysis of variance between the type of rice (brown rice and white rice) consumed and intervention (before and after) to examine the relationship between cognitive function and scores of each measurement item in the brown rice and white rice groups. In addition, we planned to conduct an exploratory analysis using path analysis to investigate the relationship between each measurement item. However, if there was a statistically significant bias in the MMSE scores between the brown rice and white rice groups at the pretest, we would have considered conducting an analysis of covariance with pre-and post-intervention differential scores for each measure as the dependent variable and with pre-intervention MMSE scores, sex, and age as adjustment factors. Furthermore, considering that the current research is a non-randomized controlled study, we planned to use the information of participants’ past medical/psychiatric history, healthy practices, dietary content other than white and brown rice, etc. as confounding factors in the analysis.

## 7. Discussions

In this intervention study, we planned to clarify the change in cognitive function, measured by several questionnaires, of the elderly by ingesting brown rice. From the results of previous studies using several types of foods including brown rice, we hypothesized that the brown rice, which contains a large amount of phenolic content and fiber content, helped maintain a higher level of cognitive function than the white rice. Normally, a random selection design is used for the intervention studies. However, it was not chosen for this study because there was no placebo material for brown rice, as the participants could easily distinguish between white and brown rice visually. In addition, considering that eating is the main activity in the daily lives of elderly people’s home residents and that rice is a staple food for Japanese people, we allowed the participants to choose their own rice diet. We assumed that this would prevent the loss of motivation that comes from being forced to be assigned to a group they did not want to be in or dropping out because their food preferences were not met. We believed that if this study could detect a cognitive maintenance effect of brown rice, it would be beneficial to examine it more closely in a future study using a randomized sampling design. Therefore, it is important to discuss the ethical considerations of staple food interventions in the future. Furthermore, considering that the intervention period of the current study is only 6 months and that the intervention method is based on only two groups of brown and white rice, in future studies, it may be worth lengthening the intervention period to measure the persistence of the effect and diversifying the food pattern to measure the synergistic effect of various nutritions.

## Figures and Tables

**Table 1:** Nutritional composition of white rice, wax-cut brown rice, and brown rice.

	White Rice	Wax-Cut Brown Rice	Brown Rice	Method
Protein (g) ^1^	5.1	5.8	6.1	Combustion method
Lipids (g)	0.8	2.6	2.9	Acid decomposition method
Ash (g)	0.3	1.1	1.2	Direct ashing method
Carbohydrate (g) ^2^	78.5	75.3	74.8	
Vitamin B1 (mg) ^3^	0.09	0.39	0.44	High performance liquid chromatography
Vitamin B2 (mg)	0.01	0.03	0.03	High performance liquid chromatography
Vitamin B6 (mg) ^4^	0.058	0.410	0.517	Microbioassay
Folic acid (μg) ^5^	18	30	29	Microbioassay
Soluble fiber (g)	-	0.2	0.2	Enzymatic-gravimetric method
Insoluble fiber (g)	0.3	2.3	2.5	Enzymatic-gravimetric method
Monounsaturated fatty acid (g)	0.14	0.76	0.90	Gas chromatography
Polyunsaturated fatty acid (g)	0.25	0.81	0.87	Gas chromatography
Oryzanol (mg)	1.2	30.1	41.4	High performance liquid chromatography
Ferulic acid (mg)	8.1	39	50	High performance liquid chromatography

Note(s): The number is the content per 100 g, calculated by Japan Food Research Laboratories. ^1^ Nitrogen/protein conversion factor: 5.95; ^2^ Calculation formula: 100 − (water + protein + lipid + ash); ^3^ As thiamine acidate; ^4^ Strain used: *Saccharomyces cerevisiae* (*S. uvarum*) ATCC 9080; ^5^ Strain used: *Lactobacillus rhamnosus* (*L. casei*) ATCC 7469.

## References

[1] Ciulu M., Cádiz-Gurrea M.L., Segura-Carretero A. (2018). Extraction and analysis of phenolic compounds in rice: A review. Molecules.

[2] Bandumula N. (2018). Rice production in Asia: Key to global food security. Proc. Natl. Acad. Sci. USA.

[3] Monika B.D. (2013). Food Security in Asia: Challenges, Policies and Implications.

[4] Liu Q., Zhou X., Sun Z. (2017). Application of silicon fertilizer affects nutritional quality of rice. Chil. J. Agric. Res..

[5] Mirtaleb S.H., Niknejad Y., Fallah H. (2021). Foliar spray of amino acids and potassic fertilizer improves the nutritional quality of rice. J. Plant. Nutr..

[6] Kozuka C., Sunagawa S., Ueda R., Higa M., Tanaka H., Shimizu-Okabe C., Ishiuchi S., Takayama C., Matsushita M., Tsutsui M. (2015). γ-Oryzanol protects pancreatic β-cells against endoplasmic reticulum stress in male mice. Endocrinology.

[7] Wang L., Lin Q., Yang T., Liang Y., Nie Y., Luo Y., Shen J., Fu X., Tang Y., Luo F. (2017). Oryzanol modifies high fat diet-induced obesity, liver gene expression profile, and inflammation response in mice. J. Agric. Food Chem..

[8] Kozuka C., Sunagawa S., Ueda R., Higa M., Ohshiro Y., Tanaka H., Shimizu-Okabe C., Takayama C., Matsushita M., Tsutsui M. (2015). A novel insulinotropic mechanism of whole grain-derived γ-oryzanol via the suppression of local dopamine D2 receptor signalling in mouse islet. Br. J. Pharmacol..

[9] Kozuka C., Yabiku K., Sunagawa S., Ueda R., Taira S., Ohshiro H., Ikema T., Yamakawa K., Higa M., Tanaka H. (2012). Brown rice and its component, γ-oryzanol, attenuate the preference for high-fat diet by decreasing hypothalamic endoplasmic reticulum stress in mice. Diabetes.

[10] Masuzaki H., Kozuka C., Okamoto S., Yonamine M., Tanaka H., Shimabukuro M. (2019). Brown rice-specific γ-oryzanol as a promising prophylactic avenue to protect against diabetes mellitus and obesity in humans. J. Diabetes Investig..

[11] Kondo K., Morino K., Nishio Y., Ishikado A., Arima H., Nakao K., Nakagawa F., Nikami F., Sekine O., Nemoto K. (2017). Fiber-rich diet with brown rice improves endothelial function in type 2 diabetes mellitus: A randomized controlled trial. PLoS ONE.

[12] Shimabukuro M., Higa M., Kinjo R., Yamakawa K., Tanaka H., Kozuka C., Yabiku K., Taira S., Sata M., Masuzaki H. (2014). Effects of the brown rice diet on visceral obesity and endothelial function: The BRAVO study. Br. J. Nutr..

[13] Mellen P.B., Walsh T.F., Herrington D.M. (2008). Whole grain intake and cardiovascular disease: A meta-analysis. Nutr. Metab. Cardiovasc. Dis..

[14] Shao Y., Bao J. (2015). Polyphenols in whole rice grain: Genetic diversity and health benefits. Food Chem..

[15] Kesse-Guyot E., Fezeu L., Andreeva V.A., Touvier M., Scalbert A., Hercberg S., Galan P. (2012). Total and specific polyphenol intakes in midlife are associated with cognitive function measured 13 years later. J. Nutr..

[16] Lamport D., Dye L., Wightman J.D., Lawton C.L. (2012). The effects of flavonoid and other polyphenol consumption on cognitive performance: A systematic research review of human experimental and epidemiological studies. Nutr. Aging.

[17] Keane K. (2017). Polyphenol Pharmacokinetics and Cardiovascular, Cognitive and Exercise Pharmacodynamics Following Montmorency Tart Cherry Intake in Humans. Ph.D. Thesis.

[18] Godos J., Caraci F., Castellano S., Currenti W., Galvano F., Ferri R., Grosso G. (2020). Association between dietary flavonoids Intake and cognitive function in an Italian cohort. Biomolecules.

[19] Godos J., Caraci F., Micek A., Castellano S., D’Amico E., Paladino N., Ferri R., Galvano F., Grosso G. (2021). Dietary phenolic acids and their major food sources are associated with cognitive status in older Italian adults. Antioxidants.

[20] Goni L., Fernández-Matarrubia M., Romanos-Nanclares A., Razquin C., Ruiz-Canela M., Martínez-González M.Á., Toledo E. (2021). Polyphenol intake and cognitive decline in the Seguimiento Universidad de Navarra (SUN) Project. Br. J. Nutr..

[21] Surh Y.J., Chun K.S., Cha H.H., Han S.S., Keum Y.S., Park K.K., Lee S.S. (2001). Molecular mechanisms underlying chemopreventive activities of anti-inflammatory phytochemicals: Down-regulation of COX-2 and iNOS through suppression of NF-κB activation. Mutat. Res.-Fund. Mol. Mech. Mutagenesis.

[22] Scott B.C., Butler J., Halliwell B., Aruoma O.I. (1993). Evaluation of the antioxidant actions of ferulic acid and catechins. Free Radic. Res. Commun..

[23] Narasimhan A., Chinnaiyan M., Karundevi B. (2015). Ferulic acid exerts its antidiabetic effect by modulating insulin-signalling molecules in the liver of high-fat diet and fructose-induced type-2 diabetic adult male rat. Appl. Physiol. Nutr. Metab..

[24] Mori T., Koyama N., Guillot-Sestier M.V., Tan J., Town T. (2013). Ferulic acid is a nutraceutical β-secretase modulator that improves behavioral impairMent. and alzheimer-like pathology in transgenic mice. PLoS ONE.

[25] Fukagawa N.K., Ziska L.H. (2019). Rice: Importance for global nutrition. J. Nutr. Sci. Vitam..

[26] Swaminathan A., Jicha G.A. (2014). Nutrition and prevention of Alzheimer’s dementia. Front. Aging NeuroSci..

[27] Lourida I., Hannon E., Littlejohns T.J., Langa K.M., Hyppönen E., Kuzma E., Llewellyn D.J. (2019). Association of lifestyle and genetic risk with incidence of dementia. JAMA.

[28] Okuda M., Fujita Y., Katsube T., Tabata H., Yoshino K., Hashimoto M., Sugimoto H. (2018). Highly water pressurized brown rice improves cognitive dysfunction in senescence-accelerated mouse prone 8 and reduces amyloid beta in the brain. BMC Complement. Altern. Med..

[29] Uenobe M., Saika T., Waku N., Ohno M., Inagawa H. (2019). Efficacy of continuous ingestion of dewaxed brown rice on the cognitive functions of the residents of elderly welfare facilities: A pilot test using crossover trial. Food Sci. Nutr..

[30] Kuroda Y., Matsuzaki K., Wakatsuki H., Shido O., Harauma A., Moriguchi T., Sugimoto H., Yamaguchi S., Yoshino O., Hashimoto M. (2019). Influence of ultra-high hydrostatic pressurizing brown rice on cognitive functions and mental health of elderly Japanese individuals: A 2-year randomized and controlled trial. J. Nutr. Sci. Vitaminol..

[31] Saika K., Yonei Y. (2021). Reduction of medical expenses by ingesting processed brown rice (sub-aleurone layer residual rinse-free rice, dewaxed brown rice). Glycative Stress Res..

[32] Sun Q., Spiegelman D., van Dam R.M., Holmes M.D., Malik V.S., Willett W.C., Hu F.B. (2010). White rice, brown rice, and risk of type 2 diabetes in US men and women. Arch. Intern. Med..

[33] Villegas R., Liu S., Gao Y.T., Yang G., Li H., Zheng W., Shu X.O. (2007). Prospective study of dietary carbohydrates, glycemic index, glycemic load, and incidence of type 2 diabetes mellitus in middle-aged Chinese women. Arch. Intern. Med..

[34] Okubo H., Inagaki H., Gondo Y., Kamide K., Ikebe K., Masui Y., Arai Y., Ishizaki T., Sasaki S., Nakagawa T. (2017). Association between dietary patterns and cognitive function among 70-year-old Japanese elderly: A cross-sectional analysis of the SONIC study. Nutr. J..

[35] Koga M., Toyomaki A., Miyazaki A., Nakai Y., Yamaguchi A., Kubo C., Suzuki J., Ohkubo I., Shimizu M., Musashi M. (2017). Mediators of the effects of rice intake on health in individuals consuming a traditional Japanese diet centered on rice. PLoS ONE.

[36] Sharif M.K., Butt M.S., Anjum F.M., Khan S.H. (2014). Rice bran: A novel functional ingredient. Crit. Rev. Food Sci. Nutr..

[37] Folstein M.F., Folstein S.E., McHugh P.R. (1975). “Mini-mental state”. A practical method for grading the cognitive state of patients for the clinician. J. Psychiatr Res..

[38] Faxen-Irving G., Andren-Olsson B., Af Geijerstam A., Basun H., Cederholm T. (2002). The effect of nutritional intervention in elderly subjects residing in group-living for the demented. Eur. J. Clin. Nutr..

[39] Salva A., Andrieu S., Fernandez E., Schiffrin E.J., Moulin J., Decarli B., Rojano-I-Luque X., Guigoz Y., Vellas B., The Nutrialz Group (2011). Health and nutrition promotion program for patients with dementia (NutriAlz): Cluster randomized trial. J. Nutr. Health Aging.

[40] Dubois B., Slachevsky A., Litvan I., Pillon B. (2000). The FAB: A Frontal assessment battery at bedside. Neurology.

[41] Guilford J.P. (1950). Creativity. Am. Psychol..

[42] Goldberg D., Williams P. (1988). A Users Guide to the General Health Questionnaire.

[43] Oba H., Sato S., Kazui H., Nitta Y., Nashitani T., Kamiyama A. (2018). Conversational assessment of cognitive dysfunction among residents living in long-term care facilities. Int. Psychogeriatr..

[44] Abete P., Della-Morte D., Gargiulo G., Basile C., Langellotto A., Galizia G., Testa G., Canonico V., Bonaduce D., Cacciatore F. (2014). Cognitive impairMent. and cardiovascular diseases in the elderly. A heart–brain continuum hypothesis. Aging Res. Rev..

[45] Foltynie T., Brayne C.E., Robbins T.W., Barker R.A. (2004). The cognitive ability of an incident cohort of Parkinson’s patients in the UK. The CamPaIGN study. Brain.

[46] Varadaraj V., Munoz B., Deal J.A., An Y., Albert M.S., Resnick S.M., Ferrucci L., Swenor B.K. (2021). Association of vision impairMent. with cognitive decline across multiple domains in older adults. JAMA Netw. Open.

[47] Dahl A., Hassing L.B., Fransson E., Berg S., Gatz M., Reynolds C.A., Pedersen N.L. (2010). Being overweight in midlife is associated with lower cognitive ability and steeper cognitive decline in late life. J. Gerontol. A Biol. Sci. Med. Sci..

[48] Bherer L., Erickson K.I., Liu-Ambrose T. (2013). A review of the effects of physical activity and exercise on cognitive and brain functions in older adults. J. Aging Res..

[49] Stubbs B., Chen L.J., Chang C.Y., Sun W.J., Ku P.W. (2017). Accelerometer-assessed light physical activity is protective of future cognitive ability: A longitudinal study among community dwelling older adults. Exp. Gerontol..

[50] Wayne P.M., Walsh J.N., Taylor-Piliae R.E., Wells R.E., Papp K.V., Donovan N.J., Yeh G.Y. (2014). Effect of Tai Chi on cognitive performance in older adults: Systematic review and meta-Analysis. J. Am. Geriatr. Soc..

[51] Yaffe K., Falvey C.M., Hoang T. (2014). Connections between sleep and cognition in older adults. Lancet Neurol..

[52] Féart C., Samieri C., Barberger-Gateau P. (2010). Mediterranean diet and cognitive function in older adults. Curr. Opin. Clin. Nutr. Metab. Care.

[53] Shatenstein B., Ferland G., Belleville S., Gray-Donald K., Kergoat M.J., Morais J., Gaudreau P., Payette H., Greenwood C. (2012). Diet quality and cognition among older adults from the NuAge study. Exp. Gerontol..

[54] Akbari E., Asemi Z., Daneshvar Kakhaki R., Bahmani F., Kouchaki E., Tamtaji O.R., Hamidi G.A., Salami M. (2016). Effect of probiotic supplementation on cognitive function and metabolic status in Alzheimer’s disease: A randomized, double-blind and controlled trial. Front. Aging NeuroSci..

[55] Sabia S., Elbaz A., Dugravot A., Head J., Shipley M., Hagger-Johnson G., Kivimaki M., Singh-Manoux A. (2012). Impact of smoking on cognitive decline in early old age: The Whitehall II cohort study. Arch. Gen. Psychiatry.

[56] Gross A.L., Rebok G.W., Ford D.E., Chu A.Y., Gallo J.J., Liang K.Y., Meoni L.A., Shihab H.M., Wang N.Y., Klag M.J. (2011). Alcohol consumption and domain-specific cognitive function in older adults: Longitudinal data from the Johns Hopkins Precursors Study. J. Gerontol. B Psychol. Sci. Soc. Sci..

[57] Zanjani F., Downer B.G., Kruger T.M., Willis S.L., Schaie K.W. (2013). Alcohol effects on cognitive change in middle-aged and older adults. Aging Ment. Health.

[58] Corley J., Jia X., Kyle J.A., Gow A.J., Brett C.E., Starr J.M., McNeill G., Deary I.J. (2010). Caffeine consumption and cognitive function at age 70: The Lothian Birth Cohort 1936 study. Psychosom. Med..

[59] Ailshire J.A., Crimmins E.M. (2014). Fine particulate matter air pollution and cognitive function among older US adults. Am. J. Epidemiol..

[60] Tsakos G., Watt R.G., Rouxel P.L., de Oliveira C., Demakakos P. (2015). Tooth loss associated with physical and cognitive decline in older adults. J. Am. Geriatr. Soc..

[61] Japan Medical Association Questionnaire on Specific Health Examination. http://amda-imic.com/oldpage/amdact/PDF/eng/spe-he-ex-e.pdf.

[62] Kumagai S., Watanabe S., Shibata H., Amano H., Fujiwara Y., Shinkai S., Yoshida H., Suzuki T., Yukawa H., Yasumura S. (2003). Effects of dietary variety on declines in high-level functional capacity in elderly people living in a community. JPN J. Public Health.

[63] Cohen S. (1988). Psychosocial models of the role of social support in the etiology of physical disease. Health Psychol..

